# Characterization and anti-salmonella activities of lactic acid bacteria isolated from cattle faeces

**DOI:** 10.1186/s12866-018-1248-y

**Published:** 2018-08-30

**Authors:** Adewale Adetoye, Eric Pinloche, Bolanle A. Adeniyi, Funmilola A. Ayeni

**Affiliations:** 1 Department of Pharmaceutical Microbiology, Faculty of Pharmacy, University of Ibadan, Ibadan, Nigeria; 2 Institute of Biological, Environmental and Rural SciencesAberystwyth University, Aberystwyth, United Kingdom

**Keywords:** Lactic acid bacteria, Cattle, Faeces, *Salmonella*, Probiotics

## Abstract

**Background:**

Non typhoidal salmonellosis is one of the neglected zoonoses in most African countries. The use of sub-therapeutic doses of antibiotics as animal growth promoter enhances the emergence and dissemination of antimicrobial resistance in bacteria with food animal reservoirs and may also results in antibiotics residue in animal products. One promising alternative to antibiotics in animal feed is Lactic Acid Bacteria (LAB) as probiotics. This study was carried out to determine the anti-salmonella activities and suitability of LAB isolated from cattle faeces in Nigeria as potential probiotics in cattle feed.

**Method:**

The test *Salmonella enterica* spp strains and LAB were isolated from cattle faeces and identified by MALDI-TOF MS and partial sequencing of 16S rRNA genes respectively. The anti-salmonella activities of the isolated LAB in co-culture, cell-free supernatant, inhibition of growth by viable LAB cells and quantification of organic acids were determined by standard techniques. The ability of the LAB strains to withstand gastric conditions, antibiotic susceptibility and their haemolytic ability on blood agar were also determined.

**Results:**

A total of 88 LAB belonging to 15 species were isolated and identified from cattle faeces. The most abundant species were *Streptococcus infantarius* (26), *Enterococcus hirae* (12), *Lactobacillus amylovorus* (10), *Lactobacillus mucosae* (10) and *Lactobacillus ingluviei* (9). Most of the LAB strains showed good anti-salmonella activities against the test *Salmonella enterica* spp. with 2 *Lactobacillus* strains; *Lactobacillus amylovorus* C94 and *Lactobacillus salivarius* C86 exhibiting remarkable anti-salmonella activities with total inhibition of *Salmonella* spp after 18 hours of co-incubation. The selected strains were able to survive simultaneous growth at pH 3 and 7% bile concentration and are non hemolytic.

**Conclusion:**

This study reports the vast diversity of culturable LAB in cattle faeces from Nigeria and their putative *in-vitro* antibacterial activity against *Salmonella enterica* spp isolated from cattle. *Lactobacillus amylovorus* C94 and *Lactobacillus salivarius* C86 demonstrated promising probiotic potentials *in-vitro* and will be further tested *in-vivo* in animal field trial.

## Background

Antibiotics resistance is a global health challenge and the causes are multifactorial with human activities being a major culprit. Antibiotics misuse and overuse in humans and livestock are major contributory factors to the emergence and transmission of antibiotics resistant organisms, the contribution of farm animals in this public health challenge is noteworthy. Growth promotion and disease prevention are important strategies in modern livestock farming; hence, there has been widespread use of antibiotics as animal feed additives [1]. The addition of such antibiotics feed additive at sub therapeutic doses for growth enhancement is a major contributing factor to the emergence and spread of antimicrobial resistant determinants among bacterial pathogens and commensals in animal reservoirs [2]. *Salmonella* is an important zoonotic pathogen [3]. *Salmonella enterica* is one of the major food borne pathogens resulting in infections ranging from acute gastroenteritis to systemic infections like typhoid fever [4]. There are about 93.8 million cases of salmonellosis in humans worldwide resulting in about 155,000 deaths annually [5]. In Africa, non-typhoidal *Salmonella* is a major cause of bacteremia particularly among children and people with impaired immune functions [6, 7] and invasive infections.

Bovine salmonellosis is also of enormous economic importance, leading to a reduction in productivity as a result of cost of treatment, weight loss, reduced meat and milk yield and mortality within the cattle herd [8]. The use of antibiotics and vaccination are some of the strategies currently being employed to combat salmonellosis [4]. However, both strategies have shortfalls while vaccination is suboptimal. The prolong use of antibiotics have a resultant effect of selecting for resistant *Salmonella* serovars and may also alter the intestinal microflora [9]. There is therefore a need for an alternative intervention against *Salmonella* infection in livestock management.

Probiotics are now being considered a promising alternative to antibiotics against enteropathogens infections [10–14]. It has been demonstrated that probiotics are useful substitutes to conventional antibiotics growth promoters especially in newly born animals [15]. Probiotics are added as feed additives to promote animal health and productivity [16]. A stable microflora of lactobacilli has been demonstrated to improve overall health performance in calves [17]. However, there is limited information on the diversity and probiotic potentials of LAB in the gut of cattle. Therefore, this study describes the diversity of culturable LAB in cattle faeces and their anti-salmonella probiotic potential *in vitro.*

## Methodology

### Samples Collection

Fresh fecal samples were collected on the ground (immediately after defecation) from 40 different cattle (Sokoto Gudali breed) , aged 2.0 ± 0.5 years at University of Ibadan Teaching and Research Dairy Farm for the isolation of LAB within a period of three months (May to July, 2015). All the cattle were certified healthy by the resident farm veterinarian. Samples collected were taken to the Pharmaceutical Microbiology laboratory for microbiological analysis within one hour of collection.

### Bacterial Isolation and Identification

#### Test Pathogens

Two *Salmonella enterica* spp designated *Salmonella enterica* S1 and *Salmonella enterica* S57 previously isolated from cattle faeces according to standard procedure [18, 19] were selected as test *Salmonella* pathogens. *Escherichia coli, Pseudomonas aeruginosa, Staphylococuus aureus* and *Klebsiella* spp from our research culture collections were also used as general test pathogens

#### Lactic Acid Bacteria

1g of cattle fecal samples were added into 9 ml of MRS broth and incubated at 37^o^C under microaerophilic condition (CampyGen^TM^ Oxoid, UK) for 24 hours, the culture were appropriately plated out on MRS agar (Oxoid, UK) and viable cells were counted. Distinct morphologically different colonies were picked from each plates and sub-cultured to obtain pure cultures. Gram positive and catalase negative isolates were preserved in 50% glycerol stock at -80^0^C.

#### Identification of the Lactic Acid Bacteria Isolates.

Identification of lactic acid bacteria in this study was done primarily by partial sequencing of 16S rRNA genes. The genomic DNA of the LAB were extracted by *AccuPrep*® Genomic DNA Extraction kit (Bioneer, South Korea) according to the manufacturer`s instruction. The extracted DNA was used as template in PCR targeted at 16S rRNA gene using the primers: 27F (AGAGTTTGATCMTGGCTCAG) and 1389R (ACGGGCGGTGTGTACAAG) with the following PCR conditions: 1 cycle of 95°C for 4 min followed with 25 cycles of 95°C for 1 min, 55°C for 1 min and 72°C for 1 min 30s and finally 1 cycle of 7 min at 72°C [20].

The PCR products obtained for 77 LAB strains were purified and sequenced. The sequences were compared with GenBank database using the basic local alignment search tool (BLAST) program for the identification of the isolates. Eleven strains whose DNA did not amplify with 16S primers were subsequently identified by MALDI-TOF MS according to standard procedure [21].

### Determination of Antimicrobial Activities of Lactic Acid Bacteria.

The anti-salmonella activities of 88 isolated viable LAB cells were carried out using a modified agar overlay method [22]. A loopful of LAB grown in MRS broth was inoculated on MRS agar plate as a line of about 2 cm long and incubated under microaerophilic condition at 37^o^C for 24 h. After incubation, the MRS agar plates were overlaid with approximately 10^5^ cfu/ml of an overnight broth culture of the two *Salmonella* test pathogens inoculated in 10 ml of Mueller Hinton (MH) soft agar (0.7% agar-agar). The overlay was allowed to set and incubated at 37^o^C under aerobic condition for 24 h and the zones of inhibition were measured.

The cell free supernatants (CFS) of all the 88 LAB isolates were further tested for antibacterial activities**.** The LAB were grown overnight in MRS broth and centrifuged at 12,000 rpm for 10 mins. One hundred μl of the CFS of the LAB strains were placed in wells (6 mm) bored into Mueller Hinton agar pre-seeded with approximately 10^5^ cfu/ml of the test *Salmonella* spp. The supernatant was allowed to diffuse for one hour before incubation at 37^o^C for 24 hrs. The plates were examined and clear zones of inhibition were measured. The antibacterial activities of seven selected LAB isolates with promising anti-salmonella activity were further determined against *Escherichia coli, Pseudomonas aeruginosa, Staphylococuus aureus* and *Klebsiella spp* in a cell free supernatant assay as described above.

Lactic acid bacteria showing promising antagonistic properties were assayed to determine the presence or absence of bacteriocin-like inhibitory substances using the agar-well diffusion method [23]. The LAB were grown in MRS broth for 18 hours and centrifuged at 12,000 rpm for 10 mins. The pellets were discarded and the pH of the cell free supernatant was adjusted to 6.2 using 1.0M NaOH. The antibacterial activities of unneutralized and neutralized CFS was tested against *Staphylococcus aureus* A104 by putting 100 μl of the CFS of the LAB strains in wells (6 mm) bored into Mueller Hinton agar pre-seeded with approximately 10^5^ cfu/ml of the test *Staphylococcus aureus*. The supernatant was allowed to diffuse for one hour before incubation at 37^o^C for 24 hrs. The plates were examined and clear zones of inhibition were measured.

### Resistance to Gastrointestinal Conditions

#### Tolerance to acidic pH

All the 88 LAB isolates were grown overnight in MRS broth under microaerophilic condition. The overnight culture was centrifuged at 12,000 rpm for 5 mins for the collection of bacterial cells. The bacterial cells were washed with sterile saline and resuspended in 10 ml fresh MRS broth and 100 μl from the culture was then inoculated into 10 ml of MRS broth which has been adjusted to pH 3.0, 4.0, 5.0 and 7.0 (with 1M HCl) and incubated at 37^o^C for 3 hours under microaerophilic condition. The initial count was done (T_0_) before incubation at 37^o^C for 3 hours under microaerophilic condition Thereafter, appropriate dilutions of the resultant culture was plated on MRS agar and incubated at 37^o^C for 24 hours under microaerophilic condition. The LAB viable count after 3 hours of contact with the modified medium was compared with the initial count.

#### Bile Tolerance

An overnight culture of all the isolated 88 LAB in MRS broth were grown at 37^o^C under microaerophilic condition and centrifuged at 12,000 rpm for 5 mins for the collection of bacterial cells. The bacterial cells were washed with sterile saline and resuspended in 10 ml fresh MRS broth. 100 μl from the culture was then inoculated into 10 ml of MRS broth supplemented with bile salt (Oxoid) to achieve 0% bile salt (control), 0.1%, 0.5%, 1%, 5% and 7 % bile concentration levels respectively. The initial count was done (T_0_) before incubation at 37^o^C for 3 hours under microaerophilic condition and incubated at 37^o^C for 3 hours under microaerophilic condition. Thereafter, appropriate dilution of the resultant culture were plated in MRS agar and incubated at 37^o^C for 24 hours under microaerophilic condition. The LAB viable count after 3 hours contact time was compared with the initial count at time 0 hour.

#### Continuous Acid and Bile Tolerance Test

Five LAB strains belonging to different *Lactobacillus* species were selected based on their antibacterial activities and resistance to gastric conditions to determine their survival in continuous acid and bile simulation. Selected LAB strains which were able to resist bile and acid separately were tested for their resistance to low pH and then bile. An overnight broth culture of LAB grown in MRS broth was centrifuged at 12,000 rpm for 10 mins for the collection of bacterial cells. The bacterial cells were washed with sterile saline and resuspended in fresh MRS broth, 100 μl from the culture was then inoculated into 10 ml of MRS broth which has been adjusted to pH 3 (with 1M HCl). The initial viable count was taken and the mixture incubated at 37^o^C for 3 hours under microaerophilic condition, after which 100 μl from the mixture were then inoculated into 10 ml of MRS broth containing 7% (w/v) bile salt and also incubated at 37^o^C for 3 hours under microaerophilic condition. The survival of the LAB were determined by plating appropriate dilution and incubating at 37^o^C under microaerophilic condition. The log reduction in the final viable LAB count in comparison with the initial count was evaluated.

### Determination of the Antibiotic Susceptibility of Lactic Acid Bacteria Isolates

As part of the European Food Safety Authority (EFSA) requirements for safety assessment of bacteria intended for probiotic purpose, such organism should not possess acquired resistance determinants to antibiotics of medical importance.

The antibiotics ampicillin, amoxicillin-clavunanic acid, vancomycin, gentamicin, kanamycin, streptomycin, erythromycin, clindamycin, tetracycline and chloramphenicol (Oxoid, UK) were tested for all the 88 isolated LAB with the disk diffusion method. A lawn of the lactic acid bacteria were made with approximately 5 x 10^7^ cfu/ml (equivalent to 0.5 McFarland standard) on *Lactobacillus* Susceptibility Test Media (LSTM). The antibiotics disc was placed on the inoculated media and incubated under microaerophilic condition at 37^o^C for 24 hours. The plates were then examined and the zones of inhibition were measured. The results were interpreted with European Committee on Antimicrobial Susceptibility Testing (EUCAST) 2016 breakpoint and the nearest species breakpoints were used for species without clearly defined breakpoints.

### Determination of Organic Acids Production by LAB

Five LAB strains belonging to different *Lactobacillus* species were selected based on their antibacterial activities and resistance to gastric conditions to determine the level of acids produced. The concentration of lactic, acetic and propionic acids produced by the selected LAB were determined using High Performance Liquid Performance Chromatography (HPLC). The HPLC system (Adept CECIL CE 4200) consisted of an HICHROM NUCLEOSIL 120-10C18 column (25cm X 4.6mm id), the column was maintained at room temperature and an aliquot (20ul) of the filtered samples was injected into the HPLC system equipped with a UV absorbance detector set at 210nm, degassed H_2_SO_4_ was used as the mobile phase. The standard curves were generated with HPLC grade lactic acid, acetic acid and propionic acid (Sigma Adreich) standards, the peak areas (mAS) were plotted against standard concentration (mg/L) to produce a standard calibration graph.

### Hemolytic Activities of LAB

Five LAB strains belonging to different *Lactobacillus* species were selected based on their antibacterial activities and resistance to gastric conditions to determine their hemolytic potential. The LAB strains were streaked on blood agar and incubated at 37^o^C for 24 hours [24]. The LAB strains that produce green-hued zones around the colonies (alpha-hemolysis) or those that do not produce any effect on the blood agar (Gamma- hemolysis) were considered non hemolytic. Those producing zones of blood lyses around the colonies are classified as hemolyic (Beta- hemolysis).

### Salmonella and Lactobacillus Co culture Experiment

Two lactic acid bacterial strains, *Lactobacillus salivarius* C86 and *Lactobacillus amylovorus* C94 were selected for *Salmonella* co culture experiments due to above average results in all the screening methods employed above. The rate of inhibition of growth of the two test *Salmonella enterica* strains by the two LAB strains were determined by a modified method of Drago *et al*. [25] in a kinetic study. A broth culture medium containing 5 ml of double strength MRS broth and 5 ml of double strength Mueller Hinton broth (MRS-MH), prepared to support the growth of both *Salmonella* and *Lactobacillus* was employed in the experiment. For the co-culture, the MRS-MH broth was inoculated with approximately 10^9^cfu/ml of LAB and 10^8^cfu/ml of the test *Salmonella enterica* spp. Two experimental controls were set up which consist of 10^9^cfu/ml of LAB as monoculture and also 10^8^cfu/ml of *Salmonella enterica* spp as monoculture. Serial dilution was carried out immediately after inoculation and appropriate dilutions of the co culture mixture were plated for time T_0_ on both MRS agar and SSA (to determine the initial counts of both organisms) at the condition of growth for each organism. LAB and *Salmonella* monoculture were plated out on MRS and SSA agar respectively This procedure was repeated every 8 hours for 24 hours, such that the cultures were serially diluted and plated out at times T_0_, T_8_, T_16_ and T_24_ hours and the viable count (cfu/ml) at each time were compared with the control grown in monoculture.

## Results

### Diversity of LAB in Bovine Faeces.

**Fig. 1:**
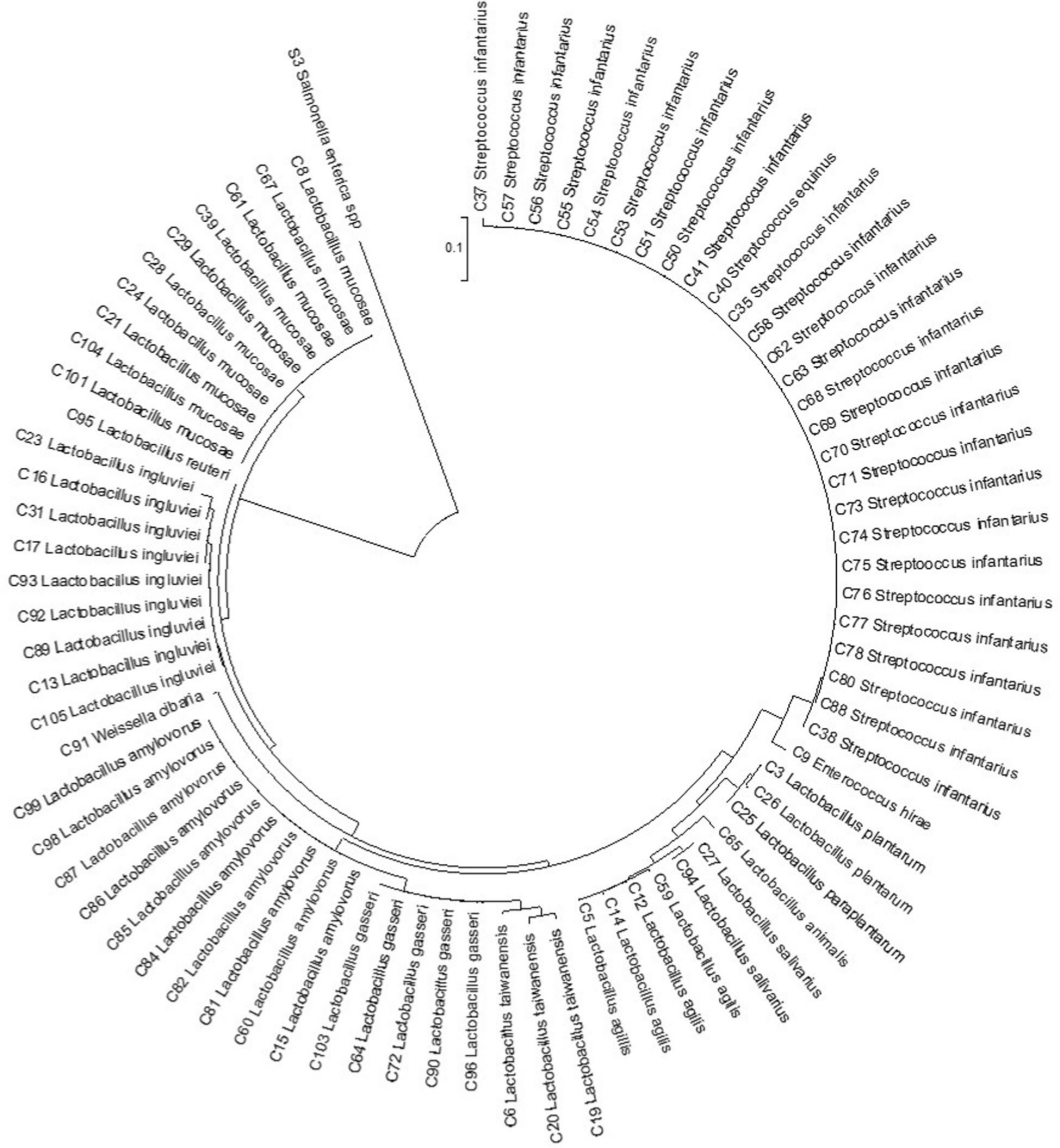
Circular phylogenetic tree based on the neighbor-joining method of 16S rRNA genes sequences of the isolated LAB and the outgroup was Salmonella enterica spp constructed using MEGA version 6. The scale bar represents 0.1-nucleotide substitutes per position

Eighty eight lactic acid bacteria were identified, belonging to 4 Genera and 15 species; *Enterococcus hirae* (12), *Lactobacillus agilis* (4), *Lactobacillus amylovorus* (10), *Lactobacillus animalis* (1), *Lactobacillus gasseri* (5), *Lactobacillus ingluviei* (9), *Lactobacillus mucosae* (10), *Lactobacillus paraplantarum* (1), *Lactobacillus plantarum* (2), *Lactobacillus reuteri* (1), *Lactobacillus salivarius* (2), *Lactobacillus taiwanensis* (3), *Streptococcus equinus* (1), *Streptococcus infantarius* (26) and *Weissella cibaria* (1) (Table 1, Fig. 1). *Streptococcus infantarius* was the most isolated species accounting for 30.68% of all the isolated LAB while *Lactobacillus animalis, Lactobacillus paraplantarum, Lactobacillus reuteri, Streptococcus equines* and *Weissella cibaria* were the least isolated with only one strain each. *Lactobacillus* (54.55%) was the most frequent genera isolated in this study. The phylogenetic relationship of the isolated lactic acid bacteria is represented in Fig. 1 showing the diversity relatedness of the different isolated species.

**Table 1:** Distribution of lactic acid bacteria isolates and their anti-salmonella activity

LAB Species	No of Isolates (%)	Zone of inhibition (mm)							
		*Salmonella enterica* S1				*Salmonella enterica* S57			
		+	++	+++	++++	+	++	+++	++++
*Lactobacillus agilis*	4 (4.55)	(0) 0	(1) 1	(2) 3	(1) 0	(0) 0	(0) 0	(4) 4	(0) 0
*Lactobacillus amylovorus*	10 (11.36)	(1) 0	(1) 2	(5) 5	(3) 3	(0) 0	(1) 2	(5) 5	(4) 3
*Lactobacillus animalis*	1 (1.14)	(0) 0	(1) 1	(0) 0	(0) 0	(0) 0	(0) 0	(1) 1	(0) 0
*Lactobacillus gasseri*	5 (5.68)	(0) 0	(1) 0	(4) 5	(0) 0	(0) 0	(2) 2	(3) 3	(0) 0
*Lactobacillus ingluviei*	9 (10.23)	(1) 1	(1) 1	(7) 7	(0) 0	(1) 1	(1) 1	(7) 7	(0) 0
*Lactobacillus mucosae*	10 (11.36)	(1) 2	(2) 2	(7) 6	(0) 0	(1) 1	(4) 4	(5) 5	(0) 0
*Lactobacillus paraplantarum*	1 (1.14)	(0) 0	(0) 0	(1) 1	(0) 0	(0) 0	(1) 1	(0) 0	(0) 0
*Lactobacillus plantarum*	2 (2.27)	(0) 0	(0) 0	(1) 2	(1) 0	(0) 0	(0) 0	(2) 2	(0) 0
*Lactobacillus reuteri*	1 (1.14)	(0) 0	(1) 1	(0) 0	(0) 0	(0) 0	(0) 1	(1) 0	(0) 0
*Lactobacillus salivarius*	2 (2.27)	(0) 0	(0) 0	(1) 1	(1) 1	(0) 0	(0) 0	(1) 1	(1) 1
*Lactobacillus taiwanensis*	3 (3.41)	(0) 0	(1) 0	(2) 3	(0) 0	(1) 1	(0) 0	(2) 2	(0) 0
*Weissella cibaria*	1 (1.14)	(0) 0	(0) 0	(1) 1	(0) 0	(0) 0	(0) 0	(1) 1	(0) 0
*Streptococcus equines*	1 (1.14)	(0) 0	(1) 1	(0) 0	(0) 0	(1) 1	(0) 0	(0) 0	(0) 0
*Enterococcus hirae*	12 (13.64)	(4) 3	(3) 4	(4) 4	(1) 1	(2) 2	(8) 8	(1) 1	(1) 1
*Streptococcus infantarius*	26 (29.55)	(4) 5	(17) 15	(3) 5	(2) 1	(3) 3	(19) 20	(4) 3	(0) 0

Diameter of zone of inhibition: 0-5 = +, >5<12= ++, 12-18 = +++, >18 = ++++. The results of cell free supernatant assay are shown in parenthesis

### Anti Microbial Activities

The anti-salmonella activities of the cell free supernatant and viable cells of the 88 isolated LAB were determined against the two test *Salmonella* strains of bovine origin. The difference between the diameters of the zones of inhibition in both assay averaged about ± 4mm with greater activities observed with the viable LAB in the agar overlay method. In both assays, the LAB isolates showed varying zones of *Salmonella* inhibition across species. Some strains of *Enterococcus hirae* and *Streptococcus infantarius* showed no activity against the test pathogens, however *Lactobacillus salivarius* C86 showed a remarkable 20 mm and 22 mm zones of inhibition, *Enterococcus hirae* 1F produced an appreciable 18mm and 20mm while *Lactobacillus amylovorus* C94 showed 21mm and 20mm zones of inhibition against *Salmonella enterica* S1 and *Salmonella enterica* S57 respectively as seen in Table 1. Based on the anti-salmonella activities, 7 LAB isolates were further tested against an array of pathogens as shown in Table 2. All the selected lactobacilli showed varying antimicrobial activities against *E. coli, S. aureus, Klebsiella* spp and *Pseudomonas aeuroginosa. Lactobacillus amylovorus* C94 and *Lactobacillus salivarius* C86 consistently exhibited the best antibacterial activities against all tested pathogens. None of the isolates tested produced bacteriocin-like inhibitory substances.

**Table 2:** Antimicrobial Activity of Selected LAB against other Pathogens

Lactic Acid Bacteria	Zones of Inhibition (mm)			
	*E. coli*	*PseudomonasAeuroginosa*	*Klebsiella spp*	*S. aureus*
*Lactobacillus plantarum C3*	12	18	14	28
*Lactobacillus amylovorus C15*	13	30	12	30
*Lactobacillus ingluviei C31*	12	12	11	28
*Lactobacillus mucosae C61*	12	20	15	30
*Lactobacillus amylovorus C86*	16	33	18	38
*Lactobacillus salivarius C94*	16	32	17	38
*Lactobacillus amylovorus C99*	15	30	14	32

### Acid and Bile Tolerance

All the tested LAB isolates were able to survive growth at the varying pH levels including the acidic pH of 3 except four *Lactobacillus* strains; *Lactobacillus* mucosae C101, *Lactobacillus ingluviei* C13*, Lactobacillus ingluviei* C89 and *Lactobacillus taiwanensis* C20 which showed no growth. The tested LAB survived the varying bile salt levels up to 5% concentration, while only six of the isolates failed to grow at 7% bile supplementation and they include; *S. infantarius* C63, *S. infantarius* 53, *S. infantarius* C78, *L. mucosae* C104, *L. mucosae* C101 and *Enterococcus hirae* C34 (results not shown). These organisms were not considered for further tests.

Both *Lactobacillus amylovorus* C94 and *Lactobacillus salivarius* C86 further demonstrated the best probiotic potentials among the selected LAB by showing considerable resistance to continuous acid and bile challenge. They were able to withstand both low pH level of 3 and simultaneous 7% bile supplementation with a 2 log_10_ reduction in cfu/ml cell count from 6.9 x 10^10^ to 7.5 x 10^8^ for *Lactobacillus salivarius* C86 and 1.9 x 10^10^ to 1.7 x 10^8^ for *Lactobacillus amylovorus* C94 as seen in Table 3.

**Table 3:** Viability of selected LAB after exposure to continuous acid and bile conditions

LAB ISOLATES	Viable count at pH 3 ( after 3 hours contact)		Viable count in Bile (after 3 hours contact)	
	initial	final	Initial	final
*Lactobacillus plantarum C3*	4.9 X 10^8^	8.9 X 10^6^	1.2 X 10^7^	1.7 X 10^5^
*Lactobacillus ingluvie C31*	2.5 X 10^10^	4.0 X 10^9^	1.3 X 10 ^8^	3.7 X 10^7^
*Lactobacillus mucosae C61*	3.4 X 10^9^	5.7 X 10^7^	8.9 X 10^6^	1.2 X 10^6^
*Lactobacillus salivarius C86*	6.9 X 10^10^	3.2 X 10^9^	1.0 X 10^9^	7.5 X 10^8^
*Lactobacillus amylovorous C94*	1.9 X 10^10^	5.7 X 10^9^	1.2 X 10^9^	1.7 X 10^8^

### Antibiotics Susceptibility of Lactic Acid Bacteria

**Fig. 2:**
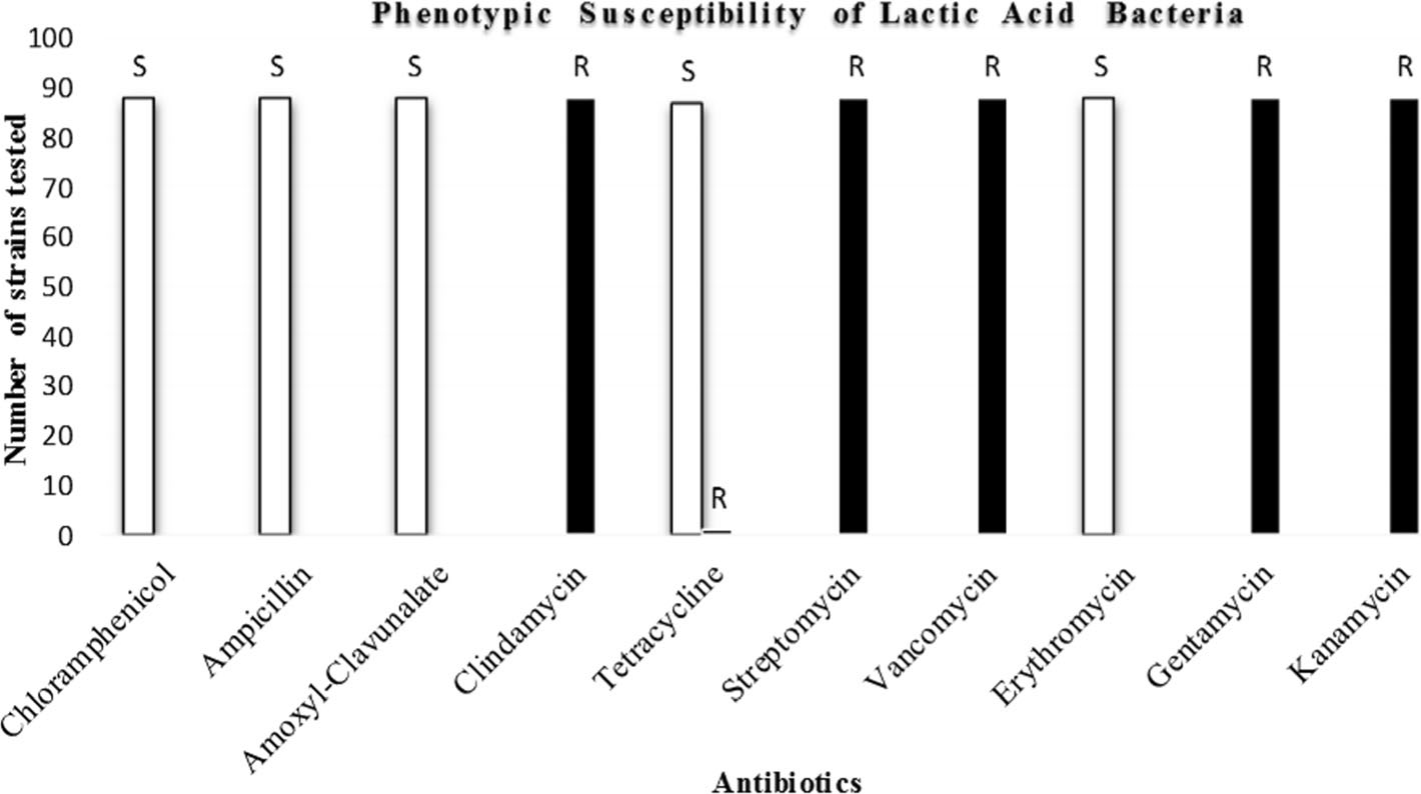
Antibiotic susceptibility pattern of the isolated lactic acid bacteria. S represents susceptible. R represents resistance

All the 88 LAB isolates were generally susceptible to chloramphenicol, ampicillin, amoxicillin-clavunalic acid and erythromycin as represented in Fig 2, there was 98.8% susceptibility to tetracycline with only one organism showing resistance, while on the other hand, there was total resistance to kanamycin, vancomycin gentamicin and clindamycin.

### Quantification of Organic Acids

**Fig. 3:**
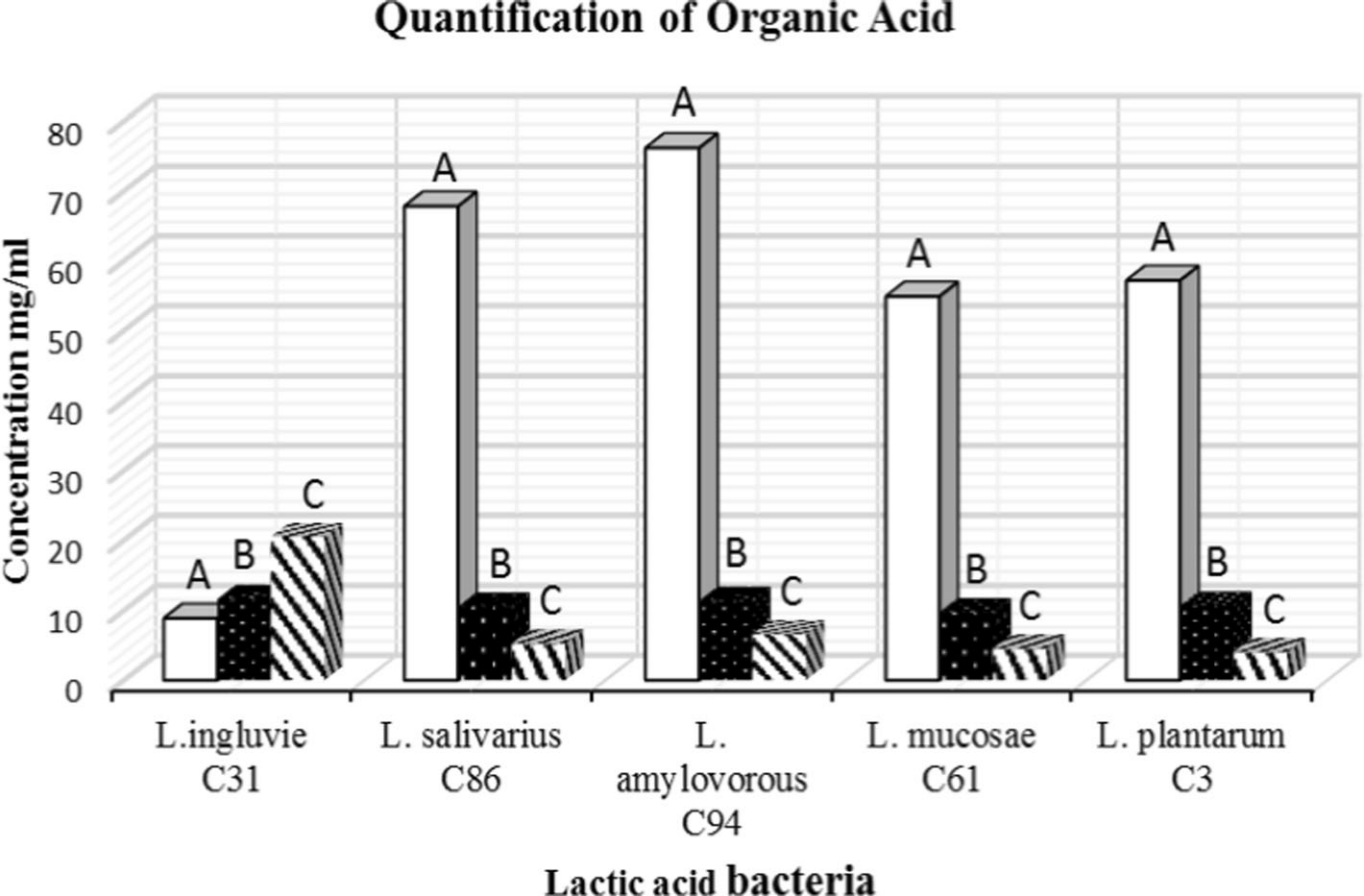
Concentration (mg/ml) of organic acid produced by selected LAB strains isolated from cattle faeces. **a** indicates yield of lactic acid. **b** indicates yield of acetic acid. **c** indicates yield of propionic acid

Generally, the concentration of lactic acid produced by all the tested strains was more than acetic acid, accounting for about 79.56% to 81.11% of all organic acid tested while propionic acid was the least produced (5.61% - 6.99%) except for *Lactobacillus ingluvie* C31 which produced mostly propionic acid (49.91%) and lactic acid was the least (21.66%) produced organic acid by this strain. *Lactobacillus salivarius* C86 produced the highest concentration of lactic acid 67.85 mg/ml (81.11%), followed by *Lactobacillus amylovorous* C94 which produced 54.91 mg/ml (80.93%) while *Lactobacillus ingluivie* C31 produced the least 8.88 mg/ml (21.66%) (Fig 3).

### Hemolytic activity of the LAB

The tested LAB did not exhibit any haemolytic effect on the blood agar

### Co-Culture kinetic study

**Fig. 4:**
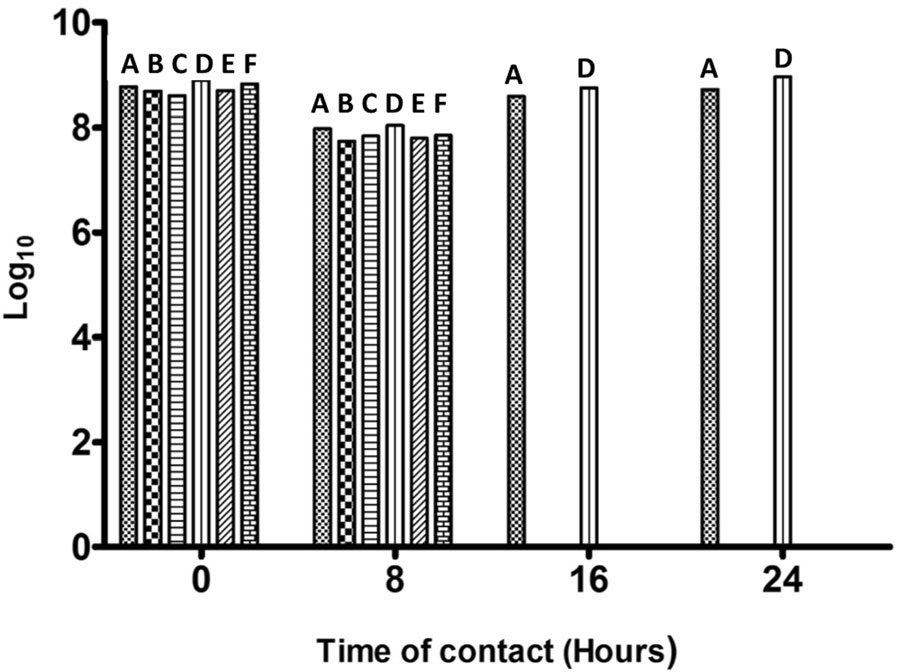
Kill Kinetics of two Salmonella enterica strains in co-culture with Lactobacillus salivarius C86 and Lactobacillus amylovorus C94. **a** represents Salmonella enterica strain S1 (control). **b** represents Salmonella enterica S1 and Lactobacillus salivarius C86. **c** represents Salmonella enterica S1 and Lactobacillus amylovorus C94. **d** represents Salmonella enterica strain S57 (control). **e** represents Salmonella enterica S57 and Lactobacillus salivarius C86. **f** represents Salmonella enterica S57 and Lactobacillus amylovorus C94

The two selected *Lactobacillus* strains; *Lactobacillus salivarius* C86 and *Lactobacillus amylovorus* C94 for co culture showed that both *Lactobacillus salivarius* C86 and *Lactobacillus amylovorus* C94 possess potent anti-salmonella activities *in vitro.* There was a drastic reduction in value from 8 log_10_ to no viable *Salmonella* cell count between 8 hours and 16 hours contact time with the two LAB strains. However, *Salmonella enterica* S1 and *Salmonella enterica* S57 groew at 3.9 x10^8^ and 5.7 x 10^8^ respectively in the *Salmonella* monoculture control at T_16_. There was no difference in the *Lactobacillus* count in the LAB-Salmonella mix for both strains as compared with the *Lactobacillus* monoculture controls (Fig 4).

## Discussion

Lactic acid bacteria are usually part of the normal flora of animals and humans. The diversity of the culturable LAB in bovine faeces isolated in MRS media in this study reveal**s** eighty eight lactic acid bacteria belonging to 15 species and 4 genera; *Lactobacillus, Weissella, Streptococcus* and *Enterococcus*. *Lactobacillus* was identified as the most frequent genera while *Streptococcus infantarius* was the most abundant species isolated in this study, followed by *Enterococcus hirae.* This is contrary to the report of Adeniyi *et al.* [23] where 94.12% of the isolated LAB from cattle faeces were *Enterococcus* spp, and no *Lactobacillus* spp was isolated. Although LAB are usual residents of the bovine gut, it is noteworthy that some LAB not commonly reported in cattle faeces were identified in this study. *L. taiwanensis* is a novel *Lactobacillus* species first isolated from cattle silage in Taiwan and named after the geographical location of sample collection [26], *Streptococcus infantarius* which was the most isolated species in our study is a predominant LAB species in African fermented dairy product of animal origin but not usually isolated from fresh milk [22, 27, 28]. *L. mucosae* is a novel pig intestinal *Lactobacillus* species first described in 2000 [29] while *Streptococcus equinus* which is predominantly of horse origin and are related to *Streptococcus bovis* commonly found in cattle faeces are often grouped together as the *S. bovis/ S. equinus* complex [30].

*Lactobacillus salivarius C86, Lactobacillus salivarius C94 and Enterococcus 1F* all demonstrated significant antibacterial activity against the two test *Salmonella enterica* S1 and *Salmonella enterica* S57 isolated from cattle faeces*.* However, only *Lactobacillus salivarius* C86 and *Lactobacillus amylovorus* C94 were selected for further characterization. While *Lactobacillus* strains have earned the “Generally Regarded as Safe” status, *Enterococcus* spp have recently emerged as one of the leading causes of nosocomial infections and bloodstream infections [31, 32]. The spread of antibiotic-resistant enterococci has also become a major public health concern worldwide [33, 34] based on the aforementioned reasons, *Enterococcus hirae* 1F was excluded from further work.

An important attribute of LAB intended for oral route of administration is the ability to survive the resistance of the gastrointestinal tract including the presence of bile salt and acidity of the gastric content [35]. The ability to withstand bile salt is an important factor for the *in vitro* selection of probiotic bacteria [22, 36]. Both *Lactobacillus salivarius* C86 and *Lactobacillus amylovorus* C94 were able to survive simultaneous low pH and bile simulation at the pH of the stomach of cattle while it receives food [37]. The survived viable LAB cells in this study are within the range of viable organisms regarded adequate to exert probiotic functions in the gut, as it has been established by various authors that the consumption of about 1.0 x 10^6^ to 1.0 x10^10^ viable cells per day is required for beneficial probiotic effects [38, 39]. The ability of these two strains to withstand gastric conditions is not very surprising considering that they were isolated from the gut of cattle and thus will have better resistance than LAB isolated from other sources. Acid tolerance and bile resistant traits of intestinal *Lactobacillus* species are thought to be evolutionary means of withstanding the host defenses and surviving transit through the gastrointestinal tract. The possession of *bsh-*1 and *bsh*-2 genes which are bile salt hydrolyze genes were found to be responsible for acid and bile tolerance in *L. salivarius* UCC118 [40].

*Lactobacillus* spp can serve as microbial barrier against intestinal pathogen through competitive exclusion of pathogen binding, modulation of host’s immune system, production of antimicrobial compounds such as organic acids (e.g., lactic acid, acetic acid, propionic acid) and proteinaceous compounds such as bacteriocins [41, 42]. One of the mechanisms of anti-salmonella activities of LAB in this study is the production of organic acids since no bacteriocin-like inhibitory substance was detected. The high antibacterial activity of *Lactobacillus salivarius* C86 and *Lactobacillus amylovorus* C94 against *Salmonella* spp and other pathogens in this study correspond with the high production of lactic acid as compared with the antimicrobial activity of other LAB strains tested. It was observed that *Lactobacillus ingluivie* C31 produced the least quantity of lactic acid and consequently had the least activity against the tested pathogens, this is in tandem with the report of many researchers who have attributed the antimicrobial activity of *Lactobacillus* spp in their various studies to the production of lactic acid which usually results in low pH [43, 44]. De-Keersmaecker *et al*., [45] reported that the anti-salmonella activity of *Lactobacillus rhamnosus* was due to accumulation of lactic acid. H’utt *et al*., [46] also reported a correlation between the pH decreases, amount of lactic acid produced, and the degree of antibacterial activity of probiotic LAB strains.

Interestingly *Lactobacillus salivarius* C86 and *Lactobacillus amylovorus* C94 in this study were able to inhibit the growth of both test *Salmonella enterica* spp completely between 8 and 16 hours of co-incubation such that no *Salmonella* spp was recoverable in the growth media. Several authors have also reported strong inhibition of *Salmonella* activities by LAB in co-culture [47–49].

The safety of LAB to be used as probiotics is also of utmost importance as the risk of dissemination of resistant genes to other microorganisms is increasing. Potential probiotic strains should not possess transferrable antibiotic resistant determinants. A major consideration is to distinguish between intrinsic and acquired resistance in probiotic organisms and this can be suggested by the comparison of antibiotic susceptibility patterns of different representative strains from each species [50]. A general susceptibility and resistant pattern was observed among species of all the isolates tested which suggest intrinsic resistance. Lactobacilli are known to exhibit a wide range of antibiotic resistance naturally, which are not transmissible and do not form a safety concern [51]. The result of our antimicrobial susceptibility testing is corroborated with the report of Maldonado and Nader-Macías [52] where the entire LAB isolated from calves faeces were all susceptible to erythromycin, ampicillin and chloramphenicol and all but one isolate was resistant to the aminoglycoside kanamycin. Intrinsic resistance to aminoglycoside antibiotics in *Lactobacillus* spp has been reported by several authors [53–57]. The resistance of *Lactobacillus* species to vancomycin has also been described as intrinsic) [50, 58]. Hoque *et al.* [59] reported a high *Lactobacillus* spp resistance to tetracycline but all *Lactobacillus* spp isolated in our study were susceptible to tetracycline. The selected strains were non haemolytic, further qualifying them as potential probiotic candidates.

## Conclusion

This study demonstrated the *in vitro* anti-salmonella ability of cattle intestinal lactic acid bacteria and their potentials to function as probiotic feed additive in livestock especially to act against salmonellosis in cattle. The two selected *Lactobacillus* strains demonstrated promising potential probiotic property *in vitro.* The strains will be further tested *in vivo* for the reduction of salmonella carriage in cattle.

## Abbreviations

16SrRNA: 16S ribosomal ribonucleic acid
CFS: Cell free supernatant
CFU/ml: Colony forming unit per millilitre
DNA: Deoxyribonucleic
Fig.: Figure
HPLC: High performance liquid chromatography
*L*.: *Lactobacillus*
LAB: Lactic acid bacteria
MALDI: Matrix-assisted laser desorption/ionization time-of-flight mass spectrometry
MH: Mueller Hinton
MRS: De Man, rogosa and sharpe
PCR: Polymerase chain reaction
*S*.: *Streptococcus*
spp.: Species
SSA: Salmonella-shigella agar
